# Navigating Diagnostic Challenges: An Uncommon Case of Jejunal Leiomyoma With Intussusception and Acute Gastrointestinal Bleeding

**DOI:** 10.7759/cureus.85198

**Published:** 2025-06-01

**Authors:** Pedro Popoutchi, Pedro Averbach, Caroline Cirenza, Marcelo Averbach

**Affiliations:** 1 Coloproctology, Hospital Sírio Libanês, São Paulo, BRA

**Keywords:** acute gastrointestinal bleeding, benign smooth muscle tumors, intussusception, leiomyoma, small bowel tumors

## Abstract

This case report aims to describe an uncommon occurrence of jejunal leiomyomas, highlighting the clinical presentations, diagnostic challenges and therapeutic interventions. A 48-year-old male patient with a history of a jejuno-jejunal intussusception treated conservatively presented two years later with dark, maroon-colored stools and a hemoglobin of 6.8 g/L. Gastrointestinal upper endoscopy and colonoscopy showed no alterations that could justify the event. Endoscopic capsule revealed an ulcerated subepithelial lesion in the proximal jejunum. Abdominal CT scan indicated a 4.6-cm mesenchymal lesion (likely gastrointestinal stromal tumor (GIST) or leiomyoma) in the jejunum. It was chosen to proceed with enterectomy due to the severity of the bleeding. At the time of the operation, the histopathological characteristics of the jejunal lesion were not definitively known but histopathology confirmed a jejunal leiomyoma. This case underscores the necessity of considering jejunal leiomyoma as a cause of acute gastrointestinal bleeding and the importance of a thorough investigation into potential underlying etiologies in adult intussusception cases. Furthermore, this case illustrates the diagnostic complexities associated with jejunal leiomyomas.

## Introduction

Leiomyomas, benign smooth muscle tumors, are rarely found in the small intestine, with jejunal leiomyomas being particularly uncommon [1]. This case report describes an unusual presentation of a jejunal leiomyoma, initially manifesting as jejuno-jejunal intussusception and later as acute gastrointestinal bleeding - a clinical scenario not typically associated with this tumor type [2, 3]. The rarity of such a presentation presents diagnostic challenges and highlights the importance of maintaining a high index of suspicion in cases of unexplained gastrointestinal bleeding. This report aims to explore the clinical, diagnostic, and therapeutic aspects of managing jejunal leiomyomas, particularly when presenting with acute bleeding, and to contribute to the limited literature on this rare condition.

## Case presentation

A 48-year-old male patient from São Paulo, Brazil, presented to the emergency department in December 2023, with a one-month history of dark maroon stools occurring twice daily, fatigue, and pallor. He denied fever, chills, nausea, vomiting, chest pain, or shortness of breath.

His medical history included a 2021 bicycle accident, resulting in multiple fractures and cranial trauma. He underwent surgical reduction of a clavicular fracture and subsequently developed nausea and vomiting, initially attributed to post-traumatic gastroparesis. An abdominal CT scan with contrast at that time revealed jejuno-jejunal intussusception, which was managed conservatively. He was also on medications for depression, attention-deficit hyperactivity disorder (ADHD), and dyslipidemia, and had a history of renal calculi. His family history included a paternal grandfather with intestinal cancer and a father with esophageal cancer.

On examination, he was hemodynamically stable and had a normal abdominal exam. Lab results showed a hemoglobin of 6.8 g/dL (reference value 11.7 to 14.9 g/dL) and a hematocrit of 23.9% (reference value 35.1% to 44.1%), compared to 16.6 g/dL five months prior. White blood cell count, renal function, electrolytes, and urinalysis were normal.

**Figure 1:**
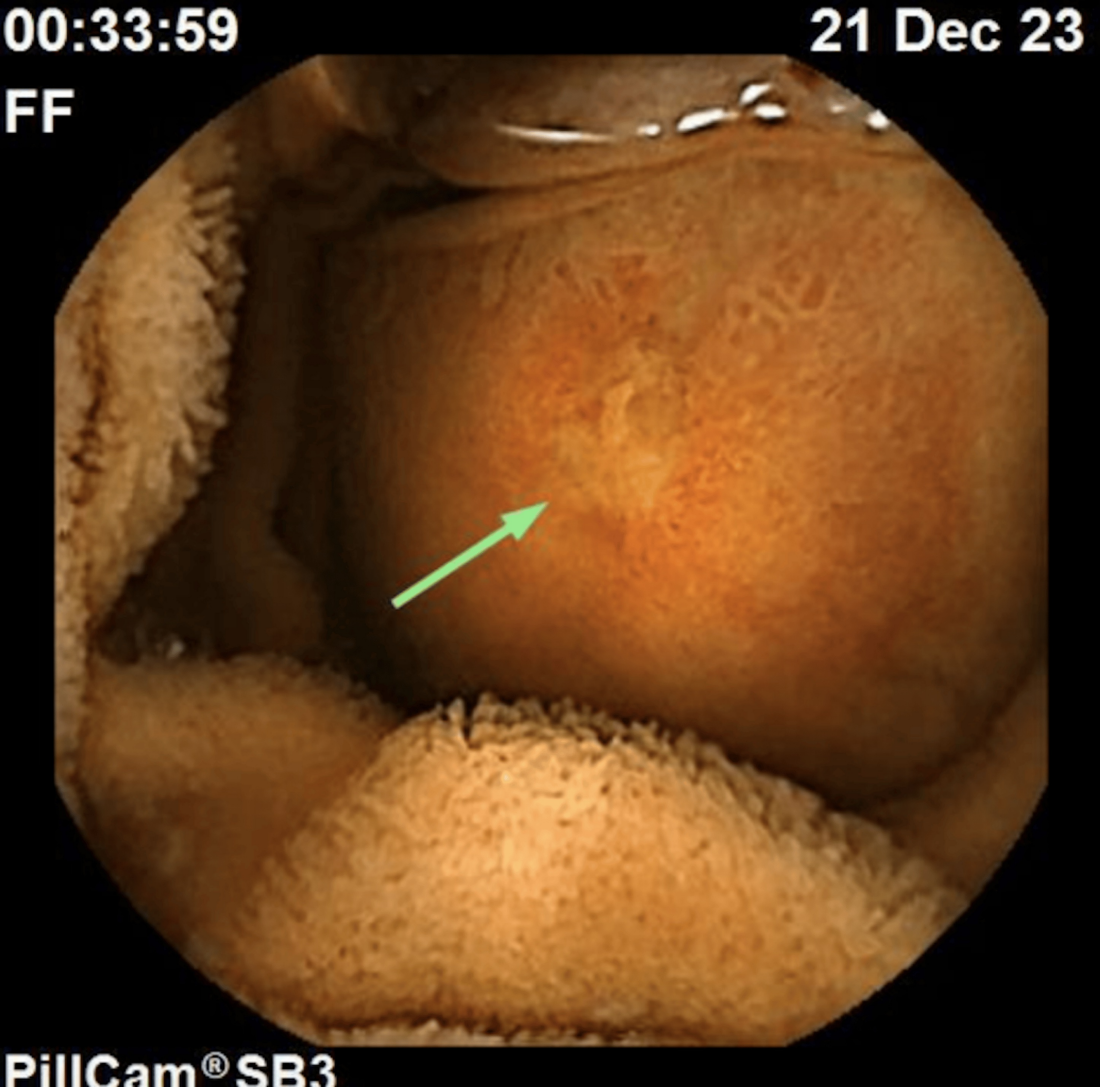
Ulcerated subepithelial lesion in the proximal jejunum.

**Figure 2:**
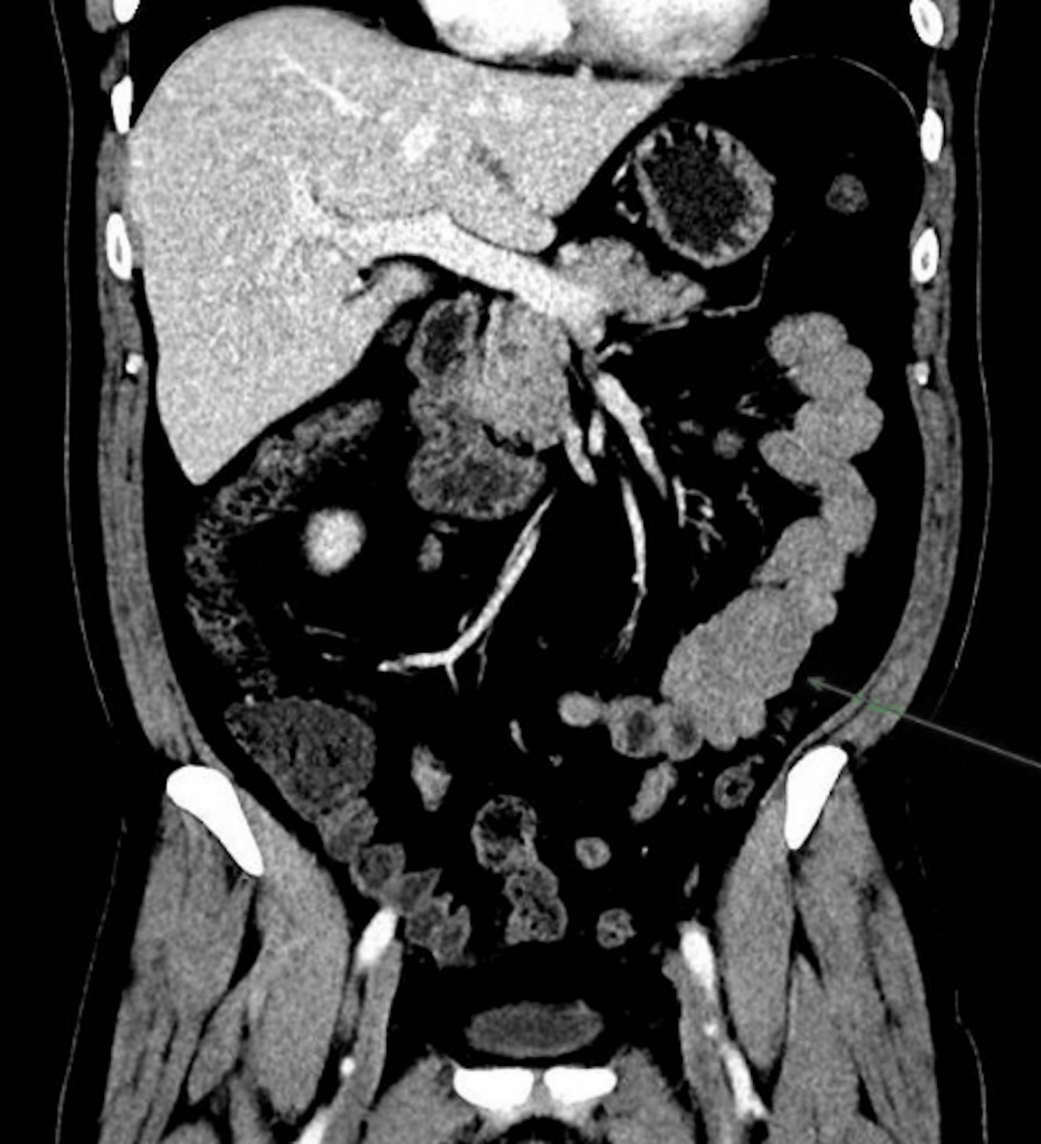
Rounded 4.6-cm jejunal nodule with well-defined limits compatible with a mesenchymal lesion (likely gastrointestinal stromal tumor (GIST) or leiomyoma).

The patient received fluids and one unit of packed red blood cells. Upper endoscopy and colonoscopy were unremarkable. Capsule endoscopy identified an ulcerated subepithelial lesion in the proximal jejunum occupying nearly the entire lumen (Figure 1). A CT scan revealed a 4.6 cm mesenchymal lesion in the jejunum, suggestive of a gastrointestinal stromal tumor (GIST) or leiomyoma (Figure 2).

**Figure 3:**
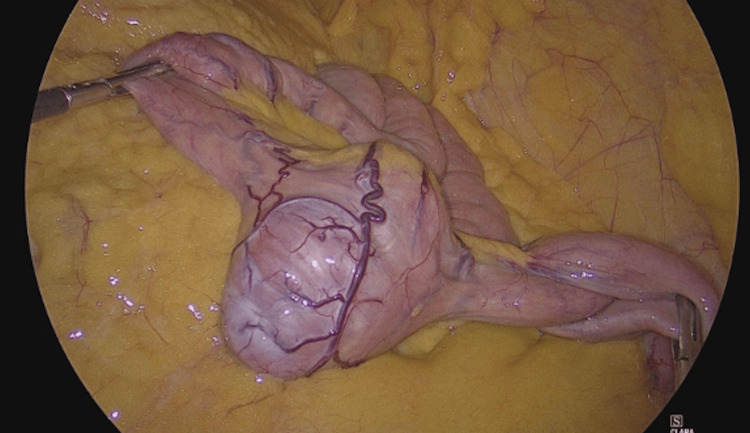
Laparoscopic view of jejunum (approximately 70 cm distal to the Treitz angle) showing a prominent, hypervascularized, round tumor measuring approximately 5x4 cm.

Over five days, the patient required five units of packed red blood cells for ongoing rectal bleeding and symptomatic anemia. Due to the severity of the bleeding (hemoglobin varied from 6.8 g/dL to 7.4 g/dL.), a laparoscopic enterectomy with extracorporeal anastomosis was performed. Intraoperatively, a hypervascular, round tumor measuring approximately 5x4 cm was found 70 cm distal to the ligament of Treitz (Figure 3). The tumor had caused a jejuno-jejunal intussusception distal to its site.

Given the urgency and uncertain histopathology at the time, an extensive dissection of the adjacent meso was performed as a precautionary oncologic measure. The patient recovered uneventfully and remains asymptomatic at follow-up.

**Figure 4:**
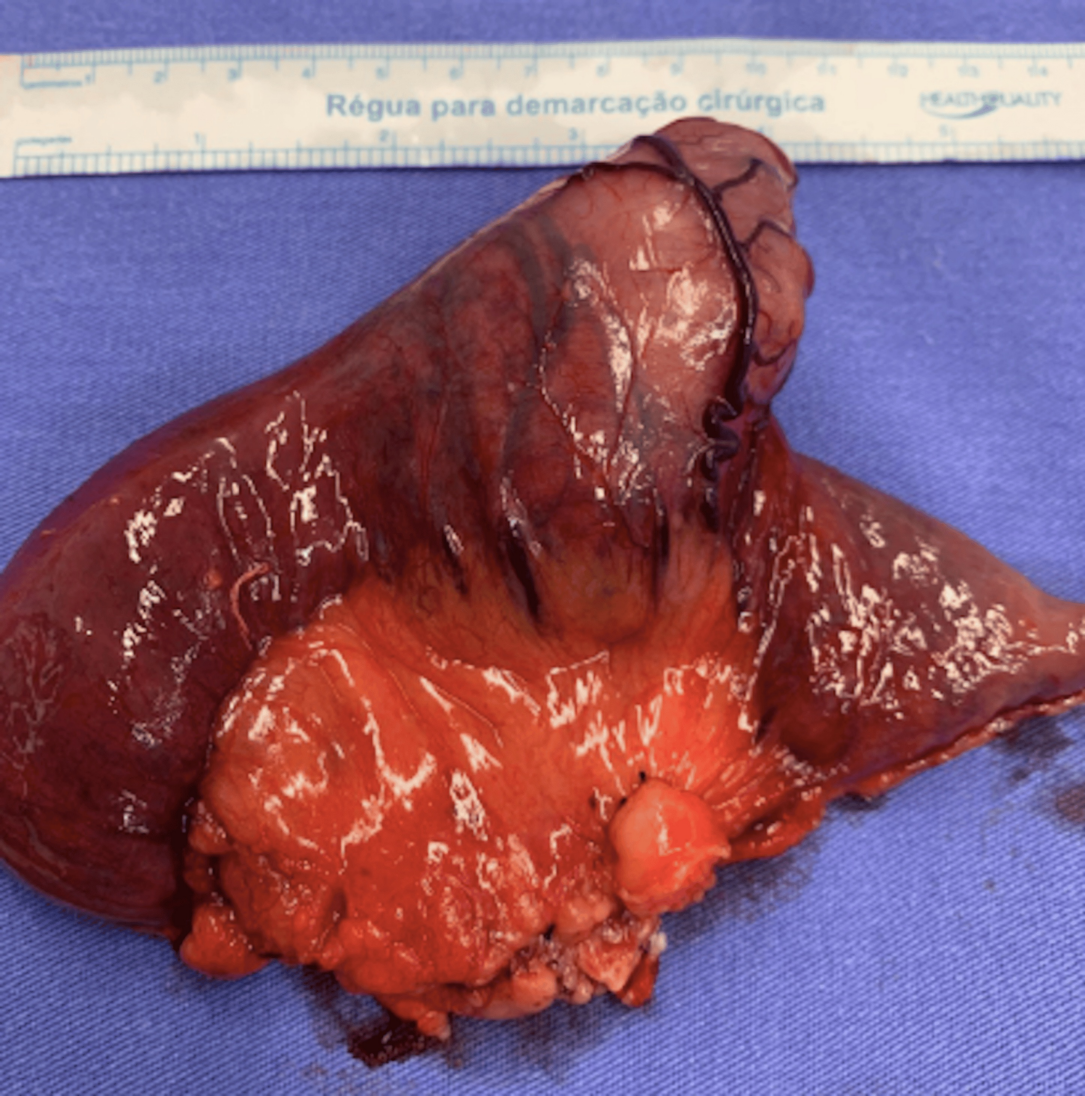
Enterectomy of the jejunum consistent with leiomyoma - nodular lesion with intramural growth and bulging of the intestinal mucosa.

**Figure 5:**
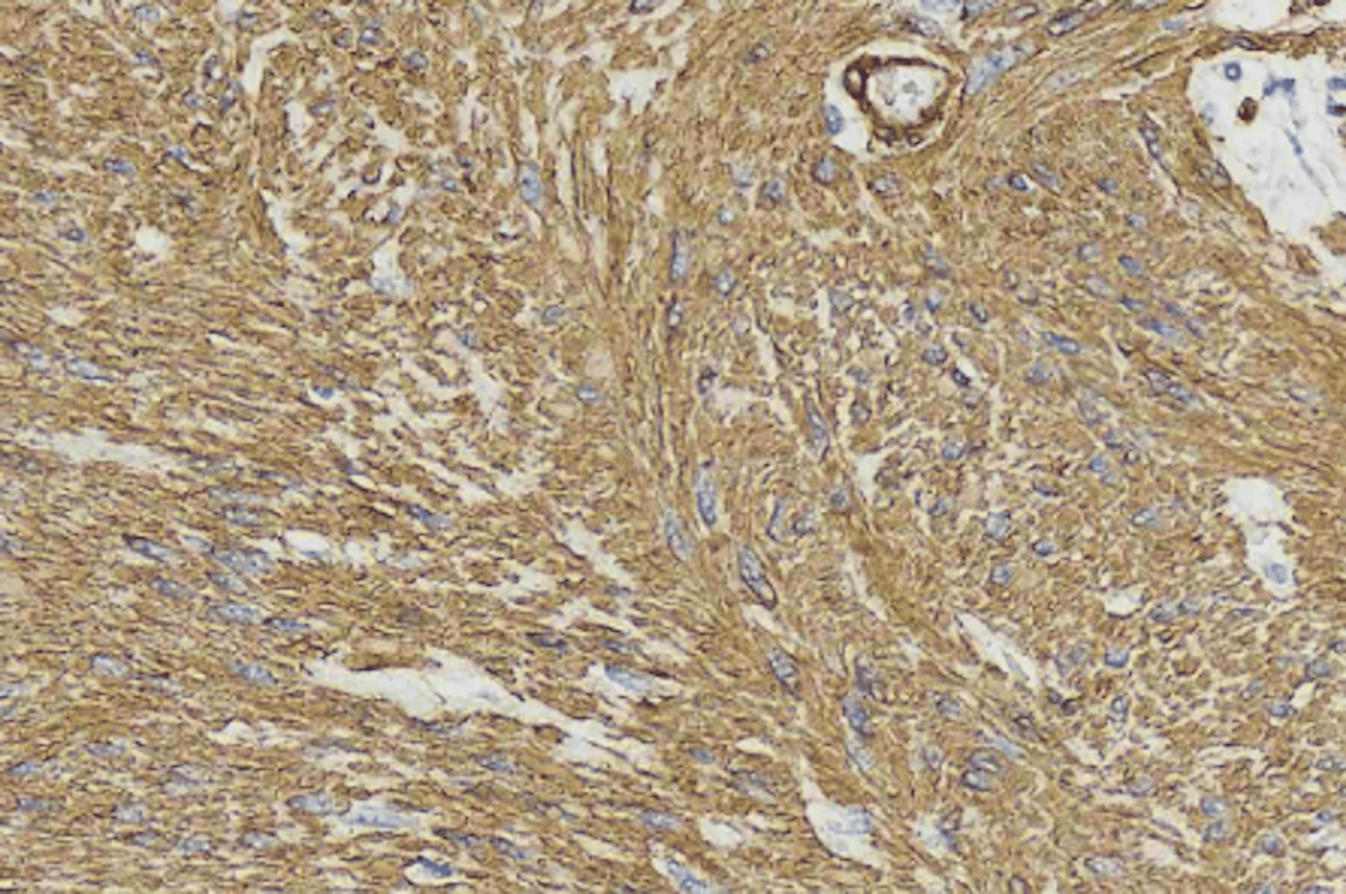
Immunohistochemistry - anti-smooth muscle actin and anti-desmin markers.

Histopathology confirmed a jejunal leiomyoma - a nodular lesion involving the muscularis mucosae, submucosa, and muscularis propria - composed of spindle cells without mitotic figures, necrosis, or hemorrhage (Figure 4). Immunohistochemistry was negative for c-KIT, DOG1, S100, and CD34, but positive for smooth muscle actin and desmin (Figure 5 and Table 1).

**Table 1:** Immunohistochemistry results. Manufacturer information: Ventana, Tucson, AZ, USA; Dako, Glostrup, Denmark.

Material	Marker	Antibody	Result
Tumor/mucosae	Anti-C-KIT	EP 10 clone / Ventana	Negative
Tumor/mucosae	Anti-DOG1	SP31 clone / Ventana	Negative
Tumor/mucosae	Anti-smooth muscle actin	HUC1-1 clone / Ventana	Positive
Tumor/mucosae	Anti-desmin	DE-R-11 clone / Ventana	Positive
Tumor/mucosae	Anti-S100	Policlonal / Ventana	Negative
Tumor/mucosae	Anti-CD34	QBEnd /10 clone / Ventana	Negative
Tumor/mucosae	Anti-Ki-67	MIB-1 clone / Dako	Positive in 1%-2% of the neoplastic cells

## Discussion

This case of jejunal leiomyoma presenting with acute gastrointestinal bleeding highlights several important aspects of small bowel tumors. The jejuno-jejunal intussusception observed in 2021, initially treated conservatively, was likely an early manifestation of the leiomyoma. In adult patients, intussusception often suggests an underlying pathology, unlike the more commonly idiopathic cases in children [4, 5].

Here, the intussusception was likely caused by the tumor serving as a lead point. This underscores the importance of a thorough diagnostic workup in adult intussusception cases. Earlier, more comprehensive imaging or endoscopic studies might have identified the tumor sooner (only CT scan was perfomerd during the intussuscepction episode).

Jejunal leiomyomas often present with nonspecific or no symptoms, making early diagnosis challenging. They are typically discovered after conservative treatments for conditions like duodenal ulcers or diverticulosis fail [3]. The acute gastrointestinal bleeding in this case deviates from the usual presentations, which more commonly involve mild bowel obstruction or pain [2].

The tumor’s hypervascularity and its role in intussusception are significant surgical findings. Only a few cases of jejuno-jejunal intussusception caused by leiomyomas have been reported in the English-language literature - seven in total, with five jejuno-jejunal and two being duodenojejunal [6-12]. This case thus contributes valuable insights into the diverse presentations and surgical implications of jejunal leiomyomas.

## Conclusions

This case underscores the diagnostic challenges posed by rare small bowel tumors like jejunal leiomyomas. It highlights the importance of including such tumors in the differential diagnosis of adult intussusception and unexplained gastrointestinal bleeding. Early and comprehensive diagnostic evaluations are critical in such atypical presentations to ensure timely and appropriate management.
